# Harrison’s Therapeutics and Materia Medica

**Published:** 1845-11

**Authors:** 


### HARRISON'S THERAPEUTICS AND MATERIA MEDICA.

The second volume of this work was placed on our table a few days ago—too late to be noticed under the head of reviews. This volume is considerably larger than the first, and evinces industry and research. Nearly two hundred pages are devoted to the consideration of bloodletting, and among other chapters we find one on enemata, one on the "anodyne or narcotic indication," and one on the "revulsive indication." In the few days that the volume has been in our possession we have found leisure for only the most cursory examination of its contents, but our inspection, slight as it has been, has impressed us with the belief that it is the better portion of the work. We hope to find time for a full notice of it in our next number. Both volumes are now on sale at the bookstores in this city. They are substantially bound, and printed in a large, clear type, on good paper. The volume before us contains upwards of 600 pages. From numerous specimens, such as the following, which met our eye as we turned over the pages of this volume, we fear that certain faults of style adverted to in our former notice, remain uncorrected. In the elaborate article on bloodletting the author says:

"Unembarrassed and confident as the medical practitioner may be as regards the propriety of vascular depletion, in cases of an ambiguous and declared inflammatory nature, yet often is he perplexed with irresolution in numerous instances of disease, in which sanguinous evacuation may be required."

No doubt of it; the physician is often in uncertainty whether he ought to bleed or not; but the fact might have been stated in somewhat fewer words. Our old preceptor, Professor Potter, was in the habit of telling us, in his lectures, to lean always to the side of the lancet in cases where there was a doubt. But we do not propose to enter upon this subject at present. Y.

